# A platelet transcriptomic signature of thromboinflammation predicts cardiovascular risk

**DOI:** 10.1172/jci.insight.195824

**Published:** 2025-12-22

**Authors:** Antonia Beitzen-Heineke, Matthew A. Muller, Yuhe Xia, Elliot Luttrell-Williams, Florencia Schlamp, Deepak Voora, Kelly V. Ruggles, Michael S. Garshick, Tessa J. Barrett, Jeffrey S. Berger

**Affiliations:** 1 Department of Medicine, New York University Grossman School of Medicine, New York, New York, USA.; 2 Department of Oncology and Hematology, University Medical Center Hamburg-Eppendorf, Hamburg, Germany.; 3 Precision Medicine in the Department of Medicine,; 4 Institute for Systems Genetics, NYU Grossman School of Medicine, New York, New York, USA.; 5 Department of Medicine, Duke University, Durham, North Carolina, USA.; 6 Sarah Ross Soter Center for Women’s Cardiovascular Research, NYU Grossman School of Medicine, New York, New York, USA.; 7 Center for the Prevention of Cardiovascular Disease, Department of Medicine, New York University Grossman School of Medicine, New York, New York, USA.

**Keywords:** Cardiology, Inflammation, Vascular biology, Atherosclerosis, Cardiovascular disease, Platelets

## Abstract

**BACKGROUND:**

Platelets are increasingly recognized as active participants in immune signaling and systemic inflammation. Upon activation, platelets form monocyte platelet aggregates (MPA) representing the crossroads of thrombosis and inflammation. We hypothesized that platelet transcriptomics could capture this thromboinflammatory axis and identify individuals at elevated cardiovascular risk.

**METHODS: MPA levels, defined as CD14+CD61+ cells, were measured using flow cytometry at 2 time points, 4 weeks apart, in healthy individuals:**

Platelets were isolated and sequenced. Individuals were categorized as MPA^hi^ or MPA^lo^ based on consistently high or low MPA levels across time points.

**RESULTS:**

Among 149 participants (median age 52 years, 57% female, 50% non-White), MPA^hi^ individuals exhibited increased expression of platelet activation markers P-selectin (*P* < 0.001), PAC-1 (*P* = 0.021), and CD40L (*P* < 0.001) and enriched immune signaling pathways. Informed by MPA levels and derived from the platelet transcriptome, we developed a 42-gene thromboinflammation platelet signature (TIPS), which correlated with MPA levels in multiple cohorts and was reproducible over time. TIPS was elevated in patients with COVID-19 (*P* = 0.0002) and myocardial infarction (*P*_adj_ = 0.008), and as in predicted future cardiovascular events in patients who underwent lower extremity revascularization after a median follow-up of 18 months (adjusted for age, sex, race, and ethnicity [adjHR] 1.55, *P* = 0.006). Notably, TIPS was modifiable by ticagrelor (*P* = 0.002) but not aspirin.

**CONCLUSION:**

These findings establish MPA as a biomarker of thromboinflammation and introduce TIPS, a platelet RNA signature, that captures thromboinflammation and provides a promising tool for cardiovascular risk stratification and a potential therapeutic target.

**TRIAL REGISTRATION:**

NCT04369664

**FUNDING:**

NIH R35HL144993, NIH R01HL139909, and AHA 16SFRN2873002 to JSB, DFG Walter-Benjamin-Programme 537070747 to AB.

## Abstract 2

Monocyte platelet aggregates are a biomarker of thromboinflammation. We created and validated a platelet RNA signature, that captures platelet-immune activity and associates with cardiovascular risk.

## Graphical abstract

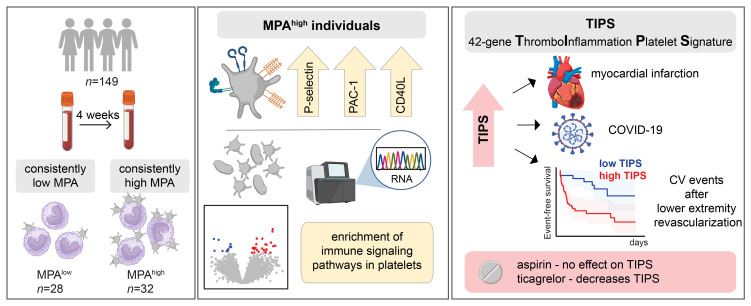

## Introduction

Platelets are key players at the intersection of atherosclerosis, thrombosis, and inflammation (1). Direct and indirect platelet-target cell interactions are fundamental in initiating and maintaining the inflammatory processes during atherothrombosis (1). Within this process, an important mechanism is the interaction of platelets and monocytes. Binding of activated platelets and monocytes occurs via P-selectin/PSGL-1 and CD40L/CD40 interaction, resulting in the formation of monocyte platelet aggregates (MPA) (2). Different platelet-derived soluble factors act as chemoattractants to monocytes and may shape monocyte phenotypes toward a proinflammatory or proreparative phenotype (3–6). MPA formation has been thought to be at the crossroad of thrombosis and inflammation (7, 8).

MPA levels represent the percent of monocytes that have platelets adherent to it and are considered a biomarker of platelet activity and their thromboinflammatory potential (9, 10). Increased MPA levels are associated with cardiovascular diseases, including coronary artery disease and peripheral artery disease (PAD) (9, 11, 12). Moreover, higher MPA levels are associated with adverse outcome in patients with acute myocardial infarction and stable cardiovascular disease (9, 13). Despite the association of MPA with cardiovascular risk factors and outcomes, its measurement and reproducibility is limited by blood collection and processing techniques (14). While platelet RNA isolation and sequencing have important limitations as well, platelet RNA-Seq is increasingly used to gain insight into platelet biology and as diagnostic and prognostic biomarker in various diseases (15, 16). Longitudinal studies demonstrate that the platelet transcriptome is stable over several years, but the platelet transcriptome can be influenced by the uptake of extracellular RNA and vesicles from the circulation and selective RNA degradation (17, 18). Platelet RNA-Seq extends beyond surface markers to reveal signaling pathways engaged during platelet-monocyte binding, offering deeper mechanistic insights into the platelet-immune crosstalk that drives vascular inflammation. By integrating cellular interactions and functional responses, a platelet transcriptomic signature of thromboinflammation could serve as a more comprehensive and potentially more sensitive tool for assessing thromboinflammation.

In the present study, we prospectively investigated the platelet phenotype (activity and transcriptome) in participants with consistently high and low MPA and demonstrated that the platelet transcriptome can identify individuals with a thromboinflammatory phenotype of individuals with high MPA. Notably, we developed a circulating genetic signature, informed by platelet-monocyte interactions and derived from an individual’s platelet transcriptome. Termed thromboinflammation platelet signature (TIPS), our signature provides a reproducible transcriptomic tool that can be used to identify individuals with and at risk of thromboinflammation.

## Results

**Figure 1:**
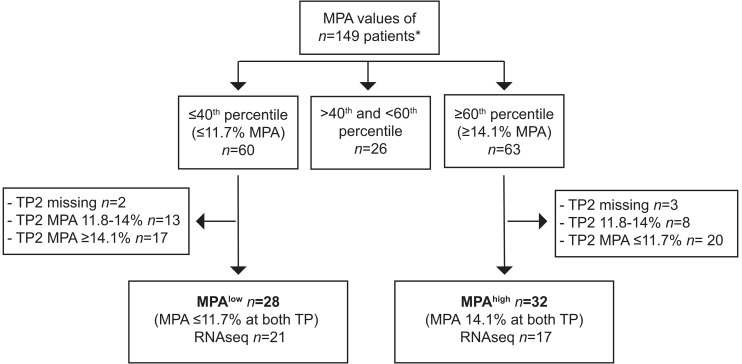
Flow chart of study participants. Percentiles of monocyte platelet aggregate (MPA) levels were calculated based on MPA values of *n* = 149 participants at 2 unique time points (TP). Participants with consistently low MPA levels (defined as ≤ 40th percentile) at 2 time points were defined as MPA^lo^ and participants with consistently high MPA levels (defined as ≥ 60th percentile) as MPA^hi^. Asterisk indicates that RNA-Seq data were available for a total of *n* = 100 participants with MPA values available at TP1 and in *n* = 102 of all study participants.

**Figure 2:**
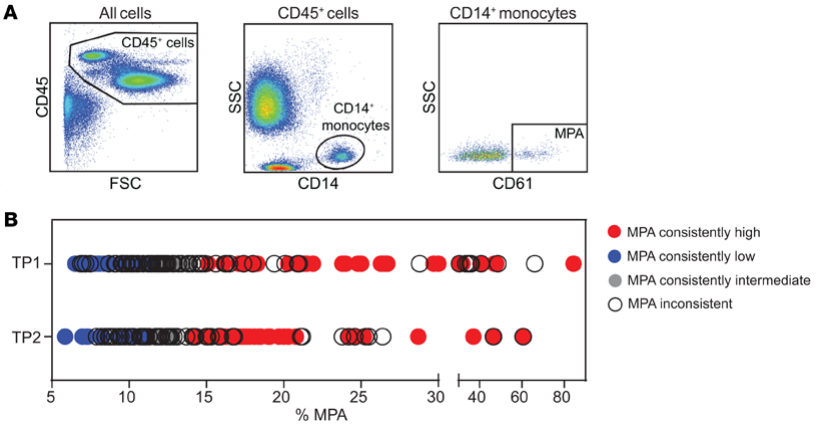
MPA gating strategy. (**A**) Using flow cytometric analysis of fixed whole blood, monocytes were identified as CD14^+^ cells within the CD45^+^ cell population. CD61^+^ cells within the CD14^+^ monocyte population were defined as monocyte-platelet aggregates (MPA). (**B**) shows MPA levels from patients with available MPA levels at both time points (TP). High MPA is defined as ≥ 14.1% and low MPA as ≤ 11.7%.

Among 149 individuals, mean age was 52 (38, 61.5) years, 57% were female and 50% were White (Table 1). Overall, the median MPA was 12.4% (10.1%, 17.7%). The median MPA did not change between time point 1 and 2 (12.5% versus 12.3%, *P* = 0.2). There was a significant, albeit modest, correlation of MPA between time points (*r* = 0.25, *P* = 0.003). To identify a consistent population with high and low MPA, we categorized individuals with MPA levels ≥ 60th percentile (14.1%) at both time points as MPA^hi^ (time point 1 and time point 2), and participants with MPA levels ≤ 40th percentile (11.7%) at both time points as MPA^lo^ (Figures 1 and 2, and Supplemental Figure 1; supplemental material available online with this article; https://doi.org/10.1172/jci.insight.195824DS1).

Baseline demographics and comorbidities of MPA^hi^ and MPA^lo^ groups are summarized in Table 1. MPA groups were not different with respect to age, race, ethnicity, or cardiovascular risk factors (BMI, smoking, family history of cardiovascular disease, hypertension, hyperlipidemia, and diabetes mellitus). In contrast, women were more frequently in the MPA^hi^ versus MPA^lo^ group (81% versus 50%, *P* = 0.022) (Table 1). After adjustment for age, sex, race, ethnicity, and BMI, only female sex was associated with higher MPA (*P* = 0.02).

Median platelet count trended higher in the MPA^hi^ versus MPA^lo^ group (264 versus 233, *P* = 0.054). No significant differences were observed for mean platelet volume, leukocytes, monocytes, or other baseline blood count parameters between groups (Table 1). The use of lipid-lowering therapy and glucose lowering medication at baseline was not different between groups (Supplemental Table 1).

### MPA is a biomarker of thromboinflammation.

**Figure 3:**
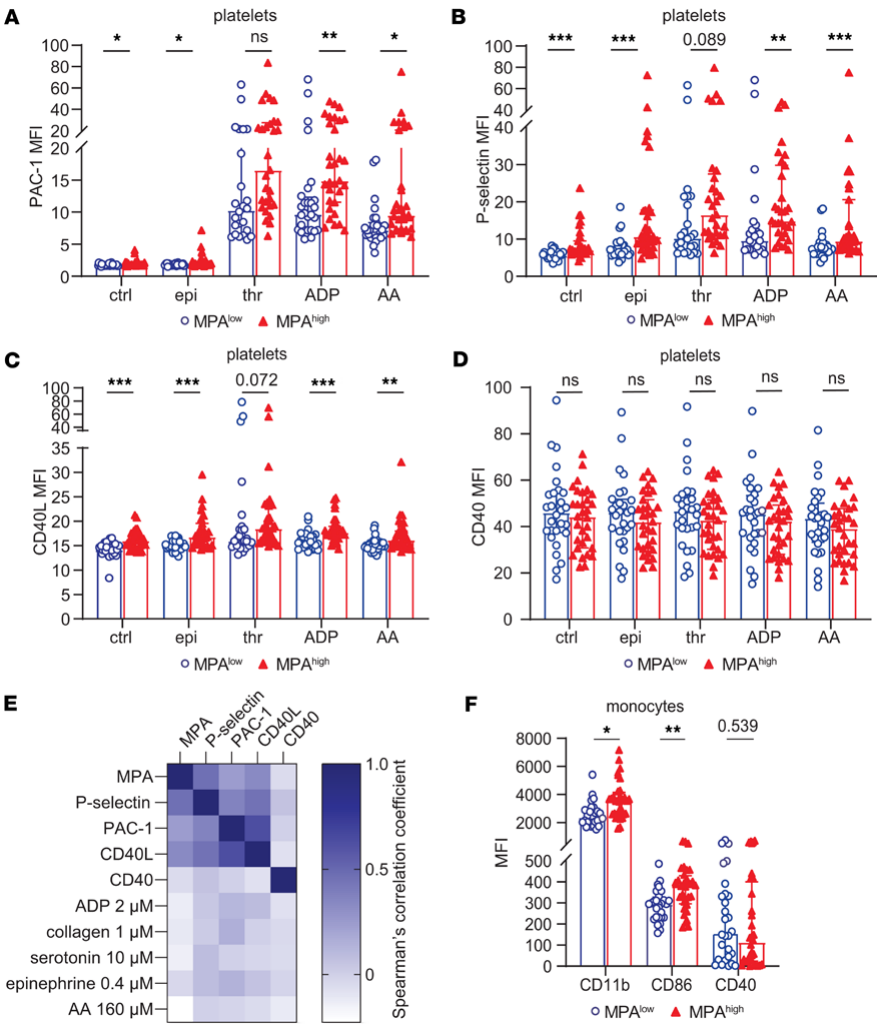
Platelet and monocyte activation markers in MPA^hi^ versus MPA^lo^ groups. Flow cytometry of whole blood was performed to analyze platelet and monocyte activation markers. (**A**–**D**) Expression of platelet activation markers PAC-1, P-selectin, CD40L, and CD40 were compared between MPA^hi^ (*n* = 32) and MPA^lo^ (*n* = 28) groups in the resting state and in the presence of the agonists epinephrine (epi), thrombin (thr), adenosine diphosphate (ADP), and arachidonic acid (AA). (**E**) Spearman’s correlation of MPA levels, platelet activation markers in the resting state measured by flow cytometry, and aggregation in response to different agonists measured by light transmission aggregometry (LTA) is shown. (**F**) Monocyte activation markers CD11b, CD86, and CD40 were measured using flow cytometry. Graphs show median and interquartile range, Man-Whitney *U* test was applied. *P < 0.05, **P < 0.01, ***P < 0.001.

Next, we investigated surface expression of platelet activation markers and monocyte activation markers between the MPA^hi^ and MPA^lo^ groups. Expression of PAC-1, P-selectin, and CD40L was significantly higher in the MPA^hi^ group compared with MPA^lo^. This difference was observed on resting platelets and after stimulation with platelet agonists epinephrine, adenosine diphosphate (ADP), and arachidonic acid (AA) (Figure 3, A–C). In contrast, platelet expression of CD40 was not different between MPA^hi^ and MPA^lo^ groups (Figure 3D). Circulating MPA levels correlated with P-selectin, PAC-1, and CD40L expression but not CD40 expression (Figure 3E).

Surface expression of monocyte activation markers CD11b and CD86 was assessed and showed higher monocyte activation in the MPA^hi^ versus MPA^lo^ group (Figure 3F). Moreover, individuals with high MPA levels also had higher levels of LPA, NPA, and LyPA compared with those with low MPA levels (Supplemental Figure 2A).

Monocytes are categorized into 3 main subpopulations: CD14^++^CD16^–^ classical monocytes, CD14^+^CD16^+^ intermediate monocytes and CD14^dim^CD16^+^ nonclassical monocytes (19). No significant differences in the monocyte subsets were found between MPA^hi^ and MPA^lo^ groups (Supplemental Figure 2, B–D).

### Platelet aggregation is not associated with MPA.

Platelet aggregation is an important measure of platelet reactivity with clinical relevance regarding CV risk (20). Platelet aggregation measured via light transmission aggregometry (LTA) using the agonists ADP, AA, collagen, serotonin, and epinephrine was not significantly different between the MPA^hi^ versus MPA^lo^ group (Supplemental Table 2) and did not significantly correlate with MPA (Figure 3E). Thus, MPA formation is a biological process that is distinct from traditional platelet aggregation responses.

### Development of TIPS.

**Figure 4:**
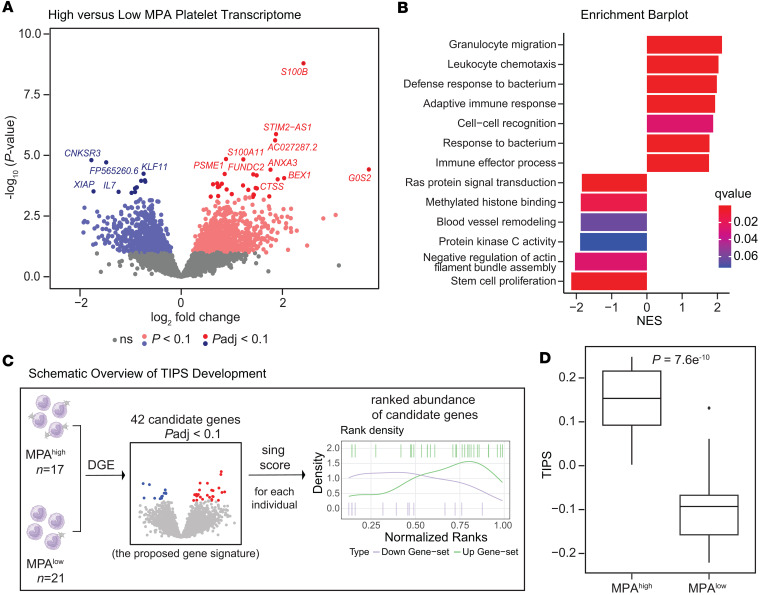
Platelet RNA transcriptomics in MPA^hi^ versus MPA^lo^ groups. Platelets were collected from healthy individuals; RNA was isolated and RNA-Seq performed. (**A**) Volcano plot showing differential gene expression (DGE), assessed using the DESeq method, in MPA^hi^ compared with MPA^lo^ groups. *P* values adjusted for age, sex, race, and ethnicity. (**B**) Gene set enrichment analysis showing differentially expressed pathways between MPA^hi^ and MPA^lo^ groups. NES, normalized enrichment score. (**C**) Based on differentially expressed genes between MPA^hi^ and MPA^lo^ groups with an adjusted *P* < 0.1 a transcriptomic signature, TIPS, was developed and calculated for each individual using the singscore method. (**D**) Box plot showing TIPS in MPA^hi^ versus MPA^lo^ individuals in the derivation cohort. Boxes show the interquartile range (IQR) with the median line, whiskers extend to 1.5 × IQR, and dots indicate outliers beyond that range. A *t* test was applied.

While platelets are anucleate, they contain RNA, which can be used to gain insights into platelet function in health and disease (15). Among the 60 individuals with consistently high or low MPA levels, 38 individuals had platelet RNA-Seq performed (*n* = 17 MPA^hi^, *n* = 21 MPA^lo^; Figure 1). Similar to the overall cohort, MPA^hi^ participants were more often female, and no significant differences were observed for age, race, ethnicity, BMI, or clinical characteristics (Supplemental Table 3). RNA-Seq revealed 29 upregulated and 13 downregulated genes (*P*_adj_ < 0.1, adjusted for multiple comparisons using the Benjamini-Hochberg method) in the platelet transcriptome between individuals with MPA^hi^ and MPA^lo^ after adjustment for age, sex, race, and ethnicity (Figure 4A and Supplemental Table 4). Top upregulated genes included genes encoding for platelet function (*FUNDC2*) (21) and genes involved in inflammation (*ANXA3* and Ca^2+^ binding protein encoding genes *S100B*, *S100A10*, *S100A11*) (22–25). Gene set enrichment analyses (GSEA) revealed biological pathways linked to leucocyte migration and chemotaxis, adaptive immunity, and response to bacteria enriched in the MPA^hi^ group, whereas pathways associated with signal transduction, cell cycle progression, and blood vessel remodeling were downregulated in the MPA^hi^ group (Figure 4B).

Using 42 genes differentially expressed (*P*_adj_ < 0.1) between high and low MPA, we developed TIPS (Figure 4C). TIPS was successful in discriminating individuals with high versus low MPA (Figure 4D). To confirm the relationship between TIPS and MPA levels, TIPS was calculated in 100 participants enrolled in the CHORD study with available platelet RNA-Seq and MPA levels at time point 1. In this cohort, there was a significant correlation between TIPS and MPA (*r* = 0.44, *P* = 0.03; Supplemental Figure 3).

### Validation of gene signature score.

To further validate the relationship between TIPS and MPA, the score was calculated in an independent cohort of women (*n* = 69, *n* = 57 with SLE, and *n* = 12 controls) with platelet RNA-Seq and MPA levels measured. Demographics for this cohort are presented in Supplemental Table 5. TIPS was significantly correlated with circulating MPA (*r* = 0.33, *P* = 0.006; Supplemental Figure 4A), confirming the relationship between TIPS and MPA.

**Figure 5:**
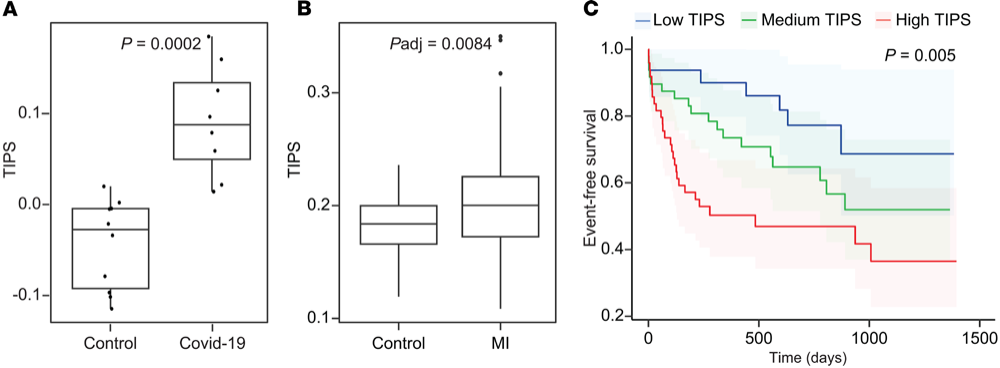
TIPS validation. TIPS was applied to cohorts with diseases associated with increased monocyte platelet aggregate (MPA) levels. (**A** and **B**) TIPS was calculated in hospitalized patients with COVID (*n* = 8) compared with matched healthy controls (*n* = 10) using a *t* test, and in 85 women undergoing coronary angiogram with myocardial infarction (MI) (*n* = 44) compared with women referred for coronary angiogram without MI (*n* = 41). Linear regression was applied and adjusted for age, race, and ethnicity as indicated. Boxes show the interquartile range (IQR) with the median line; whiskers extend to 1.5 × IQR; and points beyond that range are shown as outliers. (**C**) Among 129 patients who underwent lower extremity revascularization (LER) followed for 18 months, TIPS was calculated from platelet RNA samples collected prior to LER. Kaplan-Meyer curves of the occurrence of future cardiovascular events (composite endpoint of death, stroke, myocardial infarction, and amputation) are displayed stratified by TIPS tertiles; log-rank test was applied after adjustment for age, sex, race, and ethnicity.

Next, we investigated the clinical significance of this signature by assessing whether TIPS is associated with clinical conditions that are associated with increased MPA. In COVID-19, thromboinflammation is one of the key pathogenic mechanisms (26, 27). In a cohort of patients hospitalized with COVID-19 and matched controls (28), TIPS was significantly higher in patients with COVID-19 than controls (*P* = 0.0002; Figure 5A). In another cohort of 85 women undergoing clinically indicated coronary angiography (demographics and clinical characteristics in Supplemental Table 6) (29), TIPS was significantly higher in women with versus without an acute myocardial infarction (MI) (*P*_adj_ = 0.008, Figure 5B). Next, we investigated TIPS in patients with SLE — a disease with excess risk of thrombosis and inflammation (30–32). In a SLE cohort with platelet RNA-Seq available (Supplemental Table 7), TIPS was significantly higher in SLE compared with age and sex matched controls (*P* = 0.019; Supplemental Figure 4B). In a cohort of patients with psoriasis, an inflammatory dermatosis that is associated with an increased risk for cardiovascular disease and venous thromboembolism (33–35), there was no difference in TIPS when compared with controls (Supplemental Figure 4C and Supplemental Table 8). Consistent with this observation, MPA was higher in SLE and not in psoriasis (36, 37).

Finally, we analyzed the relationship between TIPS and high sensitive C-reactive protein (hsCRP) — a well-validated marker of inflammation that is associated with cardiovascular risk (38, 39). As noted in Supplemental Figure 5, there was no significant association between TIPS and hsCRP, suggesting that TIPS provides independent information beyond traditional markers of inflammation.

### TIPS is reproducible over a 4-week time period.

Next, we investigated the reproducibility of TIPS over time by calculating TIPS in all individuals at TP1 and TP2. Overall, there was a significant correlation of TIPS over time (*r* = 0.84, *P* < 0.001; Supplemental Figure 6A). Moreover, when individuals were stratified into tertiles of TIPS, 79% of participants who were in the lowest tertile at TP1 stayed in the lowest tertile at TP2, and 79% of participants who were in the highest tertile at TP1 stayed in the highest tertile at TP2 (Supplemental Figure 6B).

### TIPS is associated with cardiovascular outcomes.

Next, we investigated whether TIPS is associated with incident cardiovascular events in a cohort of patients with PAD undergoing lower extremity revascularization (LER). Among 129 patients who underwent LER, TIPS was calculated from platelet RNA samples collected prior to LER. When stratified into tertiles of TIPS, demographics and clinical risk factors did not differ between groups (Supplemental Table 9). After a median follow-up of 18 months, 40% of patients experienced the composite endpoint of MACLE (death, stroke, MI, and major amputation). Figure 5C shows the risk of MACLE with increasing TIPS. The differences among these curves were statistically significant (log-rank test, *P* = 0.005). Each SD increment in TIPS was associated with a 55% increase in MACLE (adjusted for age, sex, race and ethnicity adjHR] 1.55, 1.14-2.12, *P* = 0.006).

### TIPS correlates with PRESS.

**Figure 6:**
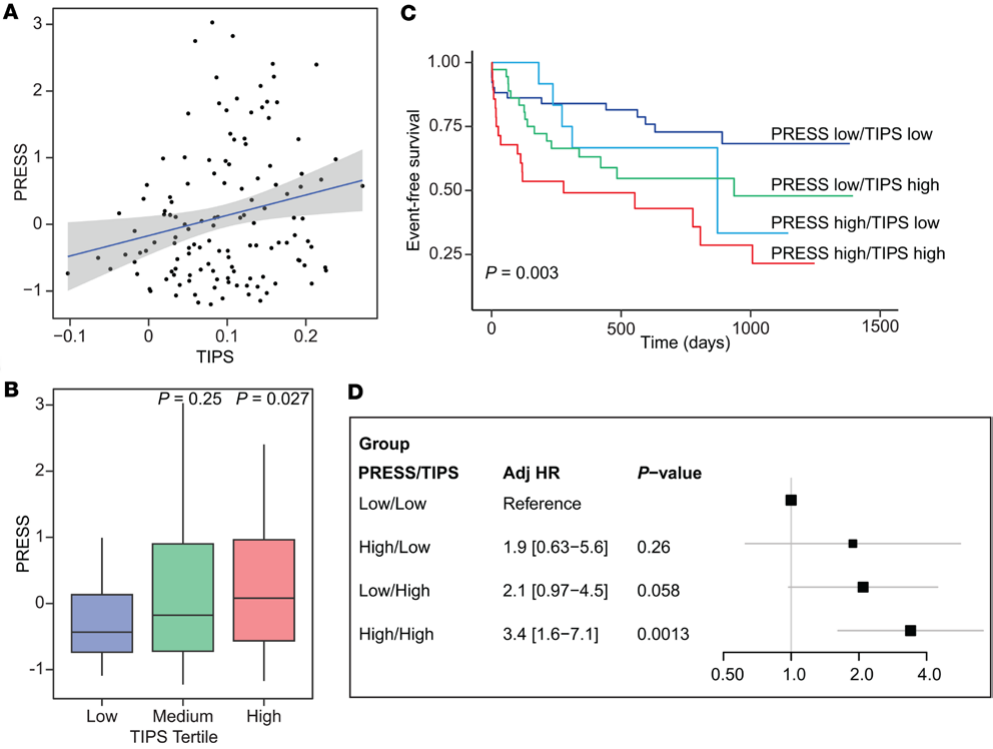
Correlation of TIPS and PRESS. TIPS and PRESS were calculated for 129 patients who underwent lower extremity revascularization (LER) followed for 18 months, from platelet RNA samples collected prior to LER. (**A**) TIPS and PRESS were correlated using Pearson’s correlation (*r* = 0.21, *P* = 0.015). (**B**) PRESS was compared between TIPS tertiles (Kruskal-Wallis). Boxes show the interquartile range (IQR) with the median line; whiskers extend to 1.5 × IQR. (**C** and **D**) Patients were stratified based on PRESS score prediction of high versus low platelet reactivity and high versus low TIPS score resulting in 4 groups: PRESS low and TIPS low (*n* = 51), PRESS high and TIPS low (*n* = 14), PRESS low and TIPS high (*n* = 36), and PRESS high and TIPS high (*n* = 28). (**C**) Kaplan-Meyer curve of risk of cardiovascular events compared between groups using log-rank test after adjustment for age, sex, race, and ethnicity. (**D**) Adjusted hazard ratios for cardiovascular event risk with low PRESS/low TIPS group as a reference.

Recently, we developed and validated the platelet reactivity expression score (PRESS) that identifies individuals with a hyperreactive platelet phenotype (platelet aggregation > 60% in response to submaximal epinephrine stimulation) and increased cardiovascular risk (40). Among the 42 genes in TIPS, only 5 (12%) were included in PRESS (*SNRPN*, *FUNDC2*, *IL7*, *NPAT*, *XIAP*). When both PRESS and TIPS were applied to the 129 patients in the PAD cohort, there was a modest correlation between the scores, suggesting independent information from each score (*r* = 0.2, *P* = 0.02; Figure 6A). PRESS was higher, albeit modestly, with increasing TIPS (*P* = 0.027) (Figure 6B). When patients were stratified by TIPS (high/low) and PRESS (high/low) into 4 groups, the combination of both scores provided independent information on risk of cardiovascular events (log-rank test, *P* = 0.003; Figure 6C). Compared with the low PRESS/low TIPS group, the group with high PRESS/high TIPS had a > 3-fold increase in MACLE (adjHR 3.4 [1.6–7.1], *P* = 0.001; Figure 6D).

### TIPS is decreased with P2Y_12_ inhibition.

**Figure 7:**
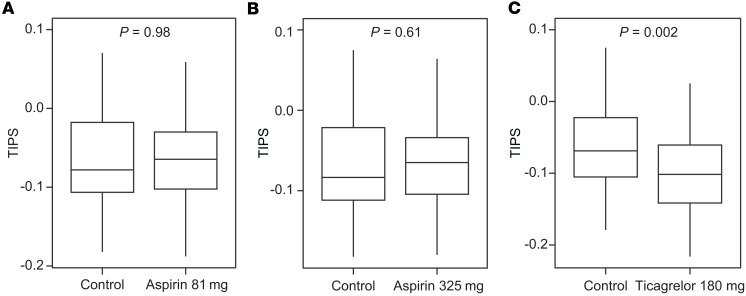
Effect of antiplatelet therapy on TIPS. (**A**–**C**) TIPS was calculated in a cohort of healthy patients that had blood collected for platelet RNA isolation at baseline (> 4 weeks without antiplatelet medication) and after 4 weeks of treatment with 81 mg aspirin (*n* = 61) (**A**), 325 mg aspirin (*n* = 62) (**B**), and 180 mg ticagrelor (*n* = 50) (**C**). Boxes show the interquartile range (IQR) with the median line; whiskers extend to 1.5 × IQR. Paired *t* test was applied.

To test whether antiplatelet therapy has an effect on TIPS, we calculated TIPS in a cohort of healthy participants who received aspirin (*n* = 62) daily or ticagrelor (*n* = 50) twice daily for 4 weeks. Blood was collected and platelet RNA was extracted before and following 4 weeks of antiplatelet therapy. Compared with baseline, TIPS did not change after 4 weeks of treatment with aspirin 81 mg (*P* = 0.98) and aspirin 325 mg (*P* = 0.61). In contrast, TIPS decreased significantly in participants taking ticagrelor 90 mg twice daily (*P* = 0.002; Figure 7, A–C). When stratified by the median baseline TIPS, ticagrelor had its greater effect in participants with elevated TIPS (Supplemental Figure 7).

## Discussion

Circulating MPA represent a biomarker of thromboinflammation and are associated with cardiovascular disease (7, 8). However, its measurement and use has been limited by blood collection and processing techniques. In this study, we overcame this limitation by investigating people with consistently high and low MPA. Our data demonstrate that consistently high MPA levels are associated with increased expression of platelet and monocyte activation markers and a platelet transcriptome enriched with immune responses and immune cell recruitment. Significantly, we developed and validated a circulating platelet genetic expression signature termed TIPS to discriminate individuals with higher MPA levels and those at increased cardiovascular risk.

This study demonstrated that high MPA levels were associated with increased expression of the activation markers P-selectin and CD40L, which are both essential surface molecules for platelet-monocyte engagement and thus MPA formation (8), as well as increased PAC-1 expression. These findings are in line with a previous, smaller study from our group that showed increased activation of platelets as measured by P-selectin and PAC-1 when comparing platelets that were aggregated to monocytes compared with nonbound platelets (9). Moreover, the present study showed increased monocyte CD11b expression, which is also involved in direct platelet-monocyte interaction (41), and CD86 expression in MPA^hi^ individuals, confirming previous data that show upregulation of these activation markers on monocytes upon MPA formation in inflammatory conditions (42, 43). While previous studies suggest preferential binding of platelets to CD16^+^ monocytes and upregulation of CD16 upon platelet monocyte interaction (9, 44–46), differences in monocyte subsets were minor between MPA^hi^ and MPA^lo^ individuals.

Platelet RNA-Seq reveals upregulation of transcripts in patients with high MPA levels that have been associated with inflammation and platelet survival and activation. One of the most upregulated transcripts (log_2_ fold change 2.42, *P* < 0.001), circulating S100B protein functions as a damage-associated molecular pattern (DAMP) and induces inflammatory responses in endothelial cells (47). The immune-proteasomal subunit PA28 (PSME1) is expressed during inflammatory conditions, and PSME1 expression was described to be increased in platelets of patients with bacterial sepsis, another condition associated with thromboinflammation (48, 49). FUNDC2 positively regulates platelet activation via AKT/GSK-3β/cGMP pathways and supports platelet survival (21, 50). Moreover, *G0S0* and *ANXA3*, which have been associated with thrombotic manifestations and cardiovascular disease, were upregulated in patients with high MPA (51–53). On the other hand, *KLF11*, which has antiinflammatory and antithrombotic effects, was downregulated in the MPA^hi^ group (54). Thus, to the best of our knowledge, this study is the first to provide platelet transcriptomic data demonstrating that MPA is a robust marker of thromboinflammation.

With platelet RNA-Seq and MPA available at 2 time points, the present study offered a unique opportunity to study healthy individuals encompassing consistently high versus low MPA levels over time, enabling the development of a platelet transcriptome signature that captures platelet-mediated thromboinflammation (TIPS). TIPS was demonstrated to be highly reproducible over time, whereas MPA levels at TP1 and TP2 only modestly correlated, suggesting that TIPS is a more robust biomarker of thromboinflammation in this study (Supplemental Figure 6). These data reinforce our rationale of selecting participants with consistently low or high MPA as the basis for the derivation of a platelet RNA signature of thromboinflammation. Moreover, TIPS was demonstrated to be increased in cohorts characterized by a heightened thromboinflammatory state, including those with cardiovascular disease as well as patients with COVID-19 and SLE, strengthening the usefulness of TIPS as a marker of thromboinflammation. Importantly, TIPS was not significantly associated with hsCRP, suggesting that TIPS likely adds independent information beyond currently used biomarkers of (vascular) inflammation.

Platelet hyperreactivity as measured by LTA is associated with increased CV risk (40). Interestingly, platelet aggregation measures did not differ significantly between MPA^hi^ and MPA^lo^ groups in the current study, suggesting that thromboinflammation (as measured by MPA) and platelet reactivity (as measured by platelet aggregation) are at least in part independent aspects of platelet biology and function. Next, we compared TIPS to the previously developed and validated platelet transcriptome signature (PRESS) that discriminates patients with platelet hyperreactivity and predicts cardiovascular risk (40). There was a small, albeit significant, correlation between TIPS and PRESS, and only a small minority of transcripts from both gene signatures overlapped, suggesting that TIPS and PRESS likely capture different aspects of the platelet phenotype and that platelet hyperreactivity (measured via platelet aggregation in response to submaximal epinephrine/PRESS) and platelet-mediated thromboinflammation (measured via MPA/TIPS) are both associated with increased CV risk. While PRESS (40) and TIPS (Figure 5C) were independently associated with cardiovascular events, when both scores were applied to the same population, subjects in the hyperreactive PRESS and high TIPS group had the highest risk of CV events, demonstrating that both scores provide independent prognostic information, suggesting a benefit of using both scores.

Finally, we investigated the effect of antiplatelet therapy on TIPS. Using platelet RNA-Seq data from a prospective study investigating aspirin and ticagrelor, we calculated TIPS in participants before and after antiplatelet therapy. Interestingly, TIPS was decreased in healthy patients after a 4-week treatment with ticagrelor compared with baseline but not in patients treated with different doses of aspirin. This is in line with previous in vitro data from our group that demonstrate inhibition of MPA formation and reduced expression of platelet-mediated proinflammatory transcripts in monocytes upon P2Y12 inhibition (55). Moreover, the highly significant decrease of TIPS in patients with baseline TIPS above the median supports the potential benefit of ticagrelor specifically in patients with elevated thromboinflammation.

Our study has several limitations. While MPA levels significantly correlated over time, reproducibility was limited (Supplemental Figure 6). While preclinical handling was standardized, minor differences in sample handling in combination with patient-specific factors might in part be responsible for inconsistent MPA levels over time. To avoid this inconsistency, we compared individuals with consistently high and low MPA, respectively. Next, while TIPS was higher in patients with acute MI, COVID-19, and SLE and associated with cardiovascular events in patients with already established cardiovascular disease and, thus, on antiplatelet therapy, long-term studies of individuals before developing cardiovascular disease could help evaluate the potential use of MPA levels or TIPS to predict cardiovascular events and, thus, influence primary preventive measures. Moreover, application of TIPS to larger cohorts of patients with different phenotypes will help validate the usefulness of TIPS as a biomarker and/or therapeutic target of cardiovascular risk. Finally, an important limitation is that absolute TIPS scores vary across different sequencing runs, and standardization will be essential before clinical application.

In summary, this study demonstrates that high MPA levels were associated with increased platelet and monocyte activation markers and a proinflammatory platelet transcriptome. We developed a platelet transcriptional signature, TIPS, that can discriminate patients with high MPA levels, is elevated in thromboinflammatory conditions, and is associated with MI and cardiovascular events. Further studies are needed to investigate the clinical use of TIPS in thromboinflammation and cardiovascular risk assessment and prevention.

## Methods

### Sex as a biological variable

Our study examined male and female patients, and sex-dimorphic effects are reported.

### Patients and blood collection

The CHOlesterol Reduction and Residual Risk in type 2 Diabetes (CHORD) study (NCT04369664) was a prospective study that investigated the effect of lipid lowering therapies in participants with LDL-C >100 mg/dL without cardiovascular disease, off all antiplatelet therapy. Blood work was performed before and after 4 weeks of lipid lowering therapy. Blood was collected without the use of a tourniquet and collected into tubes containing 3.2% sodium citrate (BD Vacutainer catalog 369714).

### Flow cytometry

Citrate-anticoagulated whole blood was fixed with 1% formalin (Crystalgen catalog CG-190), and stained with CD61 FITC (Agilent DAKO item #F0803), CD86 PE (catalog 555665), CD40 V450 (catalog 561219) (BD), CD14 PE Vio770 (catalog 130-110-521), CD45 VioGreen (catalog 130-110-638), CD16 APC Vio770 (catalog 130-113-390), CD11 APC (catalog 130-110-554), CD162 APC (catalog 130-123-841), and CD142 VioBlue (catalog 130-098-921) (Miltenyi Biotec), followed by lysis of red blood cells. MPA were identified by flow cytometry using a sequential gating strategy. First, leukocytes were gated based on CD45 expression (CD45^+^ cells). Within the CD45^+^ population, monocytes were defined as CD14^+^ cells. MPA were then identified as CD14^+^ monocytes that were also positive for CD61, indicating the presence of adherent platelets (Figure 2A). Flow cytometry was performed using a Miltenyi MACSQuant 10 (Miltenyi Biotec), and analysis was done using FlowJo software v.10.10.0. Neutrophils and lymphocytes were identified by their forward and side scatter properties and negativity for CD14.

For assessment of platelet activation markers, blood was incubated for 5 minutes with phosphate buffered saline (PBS, control) or in the presence of the agonists epinephrine (0.4 μM; catalog 5367), ADP (0.1 μM; catalog 5366), AA (160 μM; catalog 5364) (Helena Laboratories), and thrombin (0.025U; catalog 20301100) (Werfen), followed by staining with PAC1 FITC (catalog 340507), CD62p PE (catalog 555524), CD42b APC (catalog 551061), CD40 V450 (BD), and CD154 PEVio770 (Miltenyi Biotec, catalog 130-113-614). Platelets were identified based on their forward and side scatter properties and positivity for CD42b.

### Platelet aggregation

LTA was performed on a Helena Laboratories (Beaumont, TX) AggRAM based on the method of Born at 37°C under stirred conditions as previously described (40). Following 15 minutes of rest, whole blood was centrifuged at 200*g* for 10 min to obtain platelet-rich plasma (PRP). Platelet aggregation was measured in response to submaximal agonist stimulation with ADP (0.2, 1, 2 μM), AA (160, 1600 μM), collagen (0.2, 1 μg/mL; catalog 5368) (Helena Laboratories), serotonin (10μM; catalog H9523) (Millipore Sigma), and epinephrine (0.1, 0.4, 1 μM).

### RNA-Seq

Platelets were isolated using CD45 and Ter119-targeted microbeads to deplete leukocytes and red blood cells (StemCell kits, catalogs 17898 and 18170, respectively) and lysed with Qiazol lysis reagent (item #79306) followed by platelet RNA isolation with Direct-zol RNA microspin columns (Zymo Research, catalog R2062) (28, 29, 40). RNA quality and quantity were determined with a Bioanalyzer 2100 (Agilent Technologies). Sequencing libraries were barcoded and prepared using the Clontech SMART-Seq HT with Nxt HT kit (Takara Bio USA), and libraries were sequenced paired end on an Illumina NovaSeq 6000. Samples were analyzed using the Seq-N-Slide pipeline (56). Reads were aligned to the hg38 genome using STAR v2.6.1 and quantified using featureCounts v1.6.3 (57, 58). Read quality was assessed using FASTQC v0.11.7 (59). Differential expression was performed using the Wald test in DESeq2 (60).

### Development of TIPS

The platelet transcriptome was compared between the MPA^hi^ and MPA^lo^ patients who also had RNA-Seq performed. Differentially expressed genes between MPA^hi^ versus MPA^lo^ with an FDR < 0.1 after adjustment for age, sex, race, ethnicity, and after adjustment for multiple comparisons using the Benjamini-Hochberg method were identified as candidate genes that were included in TIPS. For each individual, TIPS was calculated using the upregulated and downregulated gene candidates with singscore, a rank based phenotypic scoring metric (61).

### Validation of TIPS

Platelet RNA-Seq data from multiple cohorts were used for validation of TIPS. Unless otherwise stated, subjects were recruited at New York University Langone Health and studies were approved by the New York University Grossman School of Medicine IRB.

#### COVID-19.

As previously described (62), platelet activity was measured and isolated platelets were sequenced for analysis of the platelet transcriptome in hospitalized COVID-19 patients (*n* = 8) and controls (*n* = 10).

#### Systemic lupus erythematosus (SLE).

As previously described (63), women with SLE (*n* = 121) and healthy controls (*n* = 36) had isolated platelets sequenced. Among those, 57 women with SLE and 12 healthy controls had MPA measured.

#### Psoriasis.

As previously described, participants with psoriasis (*n* = 51) and without psoriasis (*n* = 39) had platelets isolated and sequenced (36).

#### Heart attack research program (HARP).

As described previously (29), women referred for coronary angiogram with (*n* = 44) versus without MI (*n* = 41) were recruited into HARP and had their platelets isolated and sequenced.

#### Platelet activity and cardiovascular events (PACE).

As described previously (40), a cohort of 129 patients were recruited prior to LER as part of the PACE study. At baseline patients had their platelet aggregation measured and platelets isolated for RNA-Seq. Patients were followed for a median of 18 months. A composite outcome of major adverse cardiovascular and limb events (MACLE) was used for this analysis.

### Antiplatelet therapy and TIPS

As previously described (62, 64), healthy participants were recruited into a clinical study examining the effect of antiplatelet therapy on platelet activity and the platelet transcriptome. Participants received aspirin 81 mg daily, aspirin 325 mg daily, and ticagrelor 90 mg twice daily for 4 weeks. Blood was collected and platelet RNA was extracted and sequenced at baseline and following 4 weeks of therapy.

### Statistics

Categorical data are reported as numbers (percent) and continuous data as median (interquartile range [IQR]). Continuous variables were compared using the Mann Whitney *U* test for the 2-group comparison, Kruskal-Wallis was performed for the comparison among TIPS tertiles. The *χ*^2^ test or Fisher’s exact test was performed for categorical variables. Two-sided *P* < 0.05 was considered statistically significant. Continuous variables will be presented as mean ± SD or median (25th, 75th percentile), as appropriate, based on the distribution of the data.

For group comparisons of TIPS in the derivation and validation cohorts, 2-tailed *t* test or Mann-Whitney *U* test were used as indicated. For the HARP cohort, linear regression was performed adjusting for age, race, and ethnicity. Comparisons between time points were performed in a paired manner. For box-and-whisker plots, the boxes show the IQR with the median as a central line; whiskers extend to the most extreme data points within 1.5 × IQR from the quartiles (Tukey-style), and dots indicate outliers beyond this range. For correlation analyses, Pearson’s and Spearman’s correlation were used as appropriate.

Survival curves were plotted using the Kaplan-Meyer method, and statistical significance between groups was calculated using log-rank tests and cox proportional hazards models adjusted for sex, age, race and ethnicity.

### Study approval

Patients were recruited at New York University Langone Health, and the study was approved by the New York University Grossman School of Medicine IRB. Written informed consent was received prior to participation. The study examining the effect of antiplatelet therapy in healthy participants was approved by the Duke University Health System IRB, and informed consent was obtained for each patient.

### Data availability

Sequencing data are available on Gene Expression Omnibus (GEO) GSE308951. Values for graphs in the figures and supplemental figures are provided in the Supporting Data Values file. Additional data supporting the findings of this study are available from the corresponding author upon request.

## Author contributions

ABH performed research, analyzed and interpreted data, performed statistical analysis, and wrote the manuscript. MAM designed research, analyzed, and interpreted data and performed statistical analysis. YX performed statistical analysis. ELW, FS, and DV performed research and collected data. KVR contributed analytical tools and interpreted the data. MSG performed research and collected and interpreted data. TJB designed research, interpreted the data, and wrote the manuscript. JSB designed research, analyzed and interpreted data and wrote the manuscript.

## Funding support

This work is in part the result of NIH funding and is subject to the NIH Public Access Policy. Through acceptance of this federal funding, the NIH has been given a right to make the work publicly available in PubMed Central.

- American Heart Association (16SFRN2873002 to JSB)
- NIH (National Heart, Lung and Blood Institute, R35HL144993 to JSB; and NIH R01HL139909 to JSB)
- German Research Foundation (DFG, Walter Benjamin Programme, 537070747 to ABH)
- NYU Langone GTC (P30CA016087; Cancer Center Support Grant)

## Supplementary Material

Supplemental data

ICMJE disclosure forms

Supporting data values

## Figures and Tables

**Table 1:**
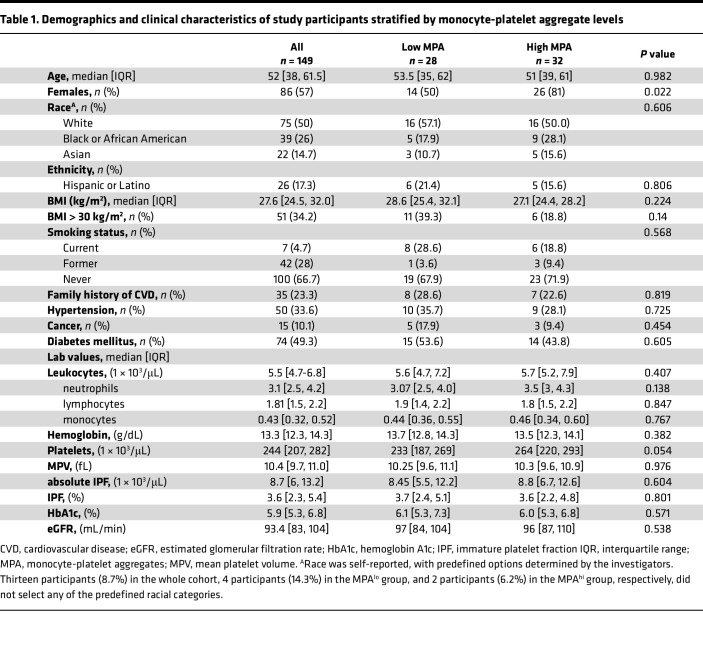
Demographics and clinical characteristics of study participants stratified by monocyte-platelet aggregate levels

## Version 1 12/22/2025

Electronic publication
