# Supplementary material for: A platelet transcriptomic signature of thromboinflammation predicts cardiovascular risk
Source: JCI Insight. 2025 Dec 22;10(24):e195824. doi: 10.1172/jci.insight.195824 (PMC12890503; doi:10.1172/jci.insight.195824)
Supplement: Supplemental data [file jciinsight-10-195824-s184.pdf]

Supplemental Material

Supplemental Table 1. Baseline medication of individuals stratified by low and high monocyte platelet aggregate (MPA) levels.

**Baseline Medications,** *n* (%)	**All** *n*=149	**MPA**^{low} *n*=28	**MPA**^{high} *n*=32	***P*-value**
**Statin**	30 (20.1)	4 (14.3)	8 (25.0)	0.349
**Ezetimibe**	4 (2.7)	2 (7.1)	0 (0.0)	0.214
**Oral antidiabetic medication**				
Metformin	47 (31.5)	11 (39.3)	9 (28.1)	0.522
SGLT2 inhibitor	12 (8.1)	5 (17.9)	2 (6.2)	0.235
DPP4 inhibitor	5 (3.4)	0 (0.0)	1 (3.1)	1
**Antidiabetic injectables**				
GLP1 agonist	29 (19.5)	7 (25.0)	4 (12.5)	0.318
Insulin	22 (14.8)	5 (17.9)	3 (9.4)	0.454

Supplemental Table 2. Platelet aggregation in participants stratified by low and high monocyte platelet aggregate (MPA) levels.

**% aggregation,** median [IQR]	**All** *n*=149	**MPA**^{low} *n*=28	**MPA**^{high} *n*=32	***P*-value**
**ADP, 2 µM**	89.2 [48.6, 92.2]	90.5 [68.1, 93.5]	85.1 [63.4, 91.6]	0.171
**ADP, 1 µM**	32.6 [15.3, 87.8]	33.6 [21.5, 84.0]	28.4 [15.3, 88.6]	0.461
**ADP, 0.2 µM**	5.6 [3.6, 10.6]	7.7 [3.8, 10.8]	5.8 [3.5, 10.1]	0.755
**AA 1600 µM**	90.4 [88.1, 92.2]	90.2 [87.3, 92.2]	89.8 [88.8, 92.3]	0.637
**AA, 160 µM**	7.7 [3.0, 89.6]	7.0 [2.4, 39.8]	36.6 [3.8, 91.3]	0.238
**collagen 1 µg/ml**	85.5 [10.7, 85.5]	85.9 [63.4, 89.6]	86.3 [63.3, 89.4]	0.951
**collagen 0.2 µg/ml**	5.7 [2.9, 9.7]	6.40 [4.0, 10.2]	5.20 [3.2, 10.4]	0.778
**serotonin 10 µM**	5.8 [4.6, 10.3]	6.25 [4.6, 10.7]	5.70 [4.6, 9.6]	0.82
**epinephrine 1 µM**	89.9 [83.4, 92.9]	90.3 [84.2, 93.9]	89.1 [79.6, 91.8]	0.447
**epinephrine 0.4 µM**	87.6 [60.0, 92.3]	87.0 [20.2, 90.1]	84.9 [40.8, 90.8]	0.72
**epinephrine 0.1 µM**	15.6 [7.4, 83.8]	15.4 [9.7, 87.7]	14.3 [7.8, 36.4]	0.273

AA, arachidonic acid; ADP, adenosine diphosphate; IQR, interquartile range; MPA, monocyte platelet aggregate.

Supplemental Table 3. Demographics and clinical characteristics of patients included in the RNAseq analysis.

	**All** *n*=102	**MPA^{low}** *n*=21	**MPA^{high}** *n*=17	***P*-value**
**Age,** median [IQR]	52 [38, 63]	52 [34.5, 62.5]	54 [39, 65]	0.706
**Females,** *n* (%)	58 (56.9)	10 (47.6)	14 (82.4)	0.057
**Race[A],** *n* (%)				0.301
White	60 (58.8)	14 (66.7)	12 (70.6)	
Black or African American	23 (22.5)	3 (14.3)	4 (23.5)	
Asian	13 (12.7)	1 (4.8)	1 (5.9)	
**Ethnicity,** *n* (%)				0.515
Hispanic or Latino	16 (15.7)	5 (23.8)	2 (11.8)	
**BMI (kg/m^2),** median [IQR]	27.7 [24.9, 31.7]	28.4 [25.4, 32.0]	27.2 [24.5, 28.3]	0.186
**BMI >30 kg/m^2,** *n* (%)	34 (33.3)	8 (38.1)	3 (17.6)	0.282
**Smoking status,** *n* (%)				0.602
Current	3 (3)	1 (4.8)	1 (5.9)	
Former	25 (24.5)	6 (28.57)	2 (11.8)	
Never	74 (72.6)	14 (66.7)	14 (82)	
**Family history of CVD,** *n* (%)	27 (26.5)	7 (33.3)	3 (17.6)	
**Hypertension,** *n* (%)	34 (33.3)	8 (38.1)	5 (29.4)	0.734
**Cancer,** *n* (%)	14 (13.7)	4 (19.0)	3 (17.6)	1
**Diabetes mellitus,** *n* (%)	52 (51)	10 (47.6)	9 (52.9)	0.945

BMI, body mass index; CVD, cardiovascular disease; IQR, interquartile range; MPA, monocyte platelet aggregate.

[A]Race and ethnicity were self-reported, with predefined options determined by the investigators. Six participants (5.9%) in the whole RNAseq cohort and 3 participants (14.3%) in the MPA^{low} group did not select any of the predefined racial categories.

Supplemental Table 4. Platelet RNA transcripts included in the Thromboinflammation Platelet Signature (TIPS).

Adjusted *P*-value <0.1	
**Upregulated**	**Downregulated**
S100B	*CNKSR3*
STIM2-AS1	*FP565260.6*
AC027287.2	*KLF11*
PSME1	*NPAT*
S100A11	*L3MBTL2-AS1*
ANXA3	*CPEB4*
G0S2	*SPEN*
FUNDC2	*GRHL1*
S100A10	*PPP4R3A*
HLA-DRA	*XIAP*
BEX1	*IL7*
CTSS	*CLASP1*
SNRPN	*HINT3*
GTF3A	
TBC1D7	
LCP1	
OSER1	
MTCH2	
AC140479.4	
POLE4	
FCER1G	
AL390728.4	
RNY3P8	
RNY3P1	
AL135938.1	
ATRAID	
NTAN1	
S100A12	
CD96	

P-value adjusted for age, sex, race, and ethnicity.

Supplemental Table 5. Demographics and clinical characteristics of patients in the SLE cohort with MPA available.

	**All** ***n*=69**
**Age,** median [IQR]	34 [27, 46]
**Race[A],** *n* (%)	
White	32 (47)
Black or African American	23 (34)
Asian	13 (19)
**Ethnicity,** *n* (%)	
Hispanic	17 (26)

IQR, interquartile range; MPA monocyte platelet aggregates; SLE, systemic lupus erythematosus.
[A]Race and ethnicity were self-reported, with predefined options determined by the investigators.

Supplemental Table 6. Demographics and clinical characteristics of patients in the HARP cohort.

	**All** ***n*=85**	**No MI** ***n*=41**	**MI** ***n*=44**	***P*-value**
**Age,** median [IQR]	61 [52, 70]	62 [57, 69]	60 [49, 72]	0.421
**Race[A],** *n* (%)				0.162
White	61 (72)	32 (78)	29 (66)	
Black or African American	14 (17)	7 (17)	7 (16)	
**Ethnicity**, *n* (%)				0.345
Hispanic or Latino	27 (32)	11 (27)	16 (36)	
**BMI (kg/m^2),** median [IQR]	27.8 [24.3, 32.1]	29.2 [24.3, 32.8]	27.3 [24.3, 32]	0.340
**Smoking status,** *n* (%)				0.605
Current	12 (14)	6 (15)	6 (15)	
Former	25 (29)	14 (34)	11 (19)	
**Hypertension,** *n* (%)	54 (64)	30 (73)	24 (55)	0.075
**Diabetes mellitus,** *n* (%**)**	29 (34)	14 (34)	15 (37)	0.996
**Hyperlipidemia,** *n* (%)	48 (54)	25 (61)	23 (44)	0.419

BMI, body mass index; IQR, interquartile range; MI, myocardial infarction; SD, standard deviation.
[A]Race and ethnicity were self-reported, with predefined options determined by the investigators. Ten participants (9%) in the whole cohort, 2 participants (5%) in the no MI group, and 8 participants (18%) in the MI group, respectively, did not select any of the predefined racial categories.

Supplemental Table 7. Demographics and clinical characteristics of patients in SLE cohort with platelet RNA sequencing available.

	**All** *n*=157	**Control** *n*=36	**SLE** *n*=121	***P*-value**
**Age,** median [IQR]	32 [25, 46]	32 [25, 46]	37 [30, 49]	0.15
**Race[A],** n (%)				0.006
White	75 (48)	19 (53)	56 (46)	
Black or African American	52 (33)	11 (31)	41 (34)	
Asian	27 (17)	3 (8)	24 (20)	
**Ethnicity**, n (%)				
Hispanic or Latino	43 (27)	7 (19)	36 (30)	0.476

IQR, interquartile range; SLE, systemic lupus erythematosus.
[A]Race and ethnicity were self-reported, with predefined options determined by the investigators. Three participants in the control group (8%) did not select any of the predefined racial categories.

Supplemental Table 8. Demographics and clinical characteristics of patients in Psoriasis cohort.

	**All** *n*=90	**Control** *n*=39	**Psoriasis** *n*=51	***P*-value**
**Age,** median [IQR]	47 [33, 60]	35 [28, 63]	47[38, 61]	0.12
**Male sex, n (%)**	51 (57)	18 (46)	33 (65)	0.08
**White[A],** n (%)	68 (76)	28 (72)	40 (78)	0.21
**Hispanic or Latino**, n (%)	16 (18)	6 (15)	10 (20)	0.6

IQR, interquartile range.
[A] Race and ethnicity were self-reported, with predefined options determined by the investigators.

Supplemental Table 9. Demographics and clinical characteristics of patients in the PACE-PAD cohort depending on TIPS tertile.

	**All** *n*=129	**Tertile 1** *n*=32	**Tertile 2** *n*=48	**Tertile 3** *n*=49	***P*-value**
**Age,** median [IQR]	70 [65, 79]	72 [67, 80]	71 [1, 79]	70 [65, 77]	0.845
**Females,** n (%)	45 (35)	13 (41)	12 (25)	20 (41)	0.193
**Race,** n (%)					0.065
White	82 (64)	28 (88)	29 (60)	25 (51)	
Black or African American	26 (20)	2 (6)	11 (23)	13 (27)	
Asian	3 (2)	0 (0)	1 (2)	2 (4)	
**Ethnicity,** n (%)					0.255
Hispanic or Latino	25 (19)	3 (9)	11 (23)	11 (22)	
**BMI (kg/m^2),** mean (SD)	26.9 (5.7)	27.1 (4.5)	27.3 (6.5)	26.3 (5.6)	0.677
**Smoking status,** n (%)					0.416
Current	19 (15)	6 (18.8)	4 (8.3)	9 (18)	
Former	79 (61)	20 (63)	33 (69)	26 (53)	
Never	31 (24.0)	6 (18.8)	11 (22.9)	14 (29)	
**Hypertension,** n (%)	109 (85)	29 (91)	39 (81)	41 (84)	0.515
**Diabetes mellitus,** n (%)	70 (54)	12 (38)	29 (60)	29 (60)	0.089
**Hyperlipidemia,** n (%)	88 (68)	21 (66)	35 (73)	32 (65)	0.677
**Antiplatelet medication,** n (%)	118 (92)	31 (97)	44 (92)	43 (88)	0.356
Aspirin	111 (86)	30 (94)	42 (88)	39 (80)	0.186
Clopidogrel	49 (38)	10 (31)	20 (42)	19 (39)	0.636
**HbA1c,** (%)	6.9 (1.4)	6.5 (1.1)	7.4 (1.5)	6.7 (1.5)	0.081

BMI, body mass index; IQR, interquartile range; PAD, peripheral artery disease; SD, standard deviation.
[A]Race and ethnicity were self-reported, with predefined options determined by the investigators. Eighteen patients (14%) in the whole cohort, 2 patients (6%) in Tertile 1, 7 patients (14.6%) in Tertile 2, and 9 patients (18%) in Tertile 3, respectively, did not select any of the predefined racial categories.

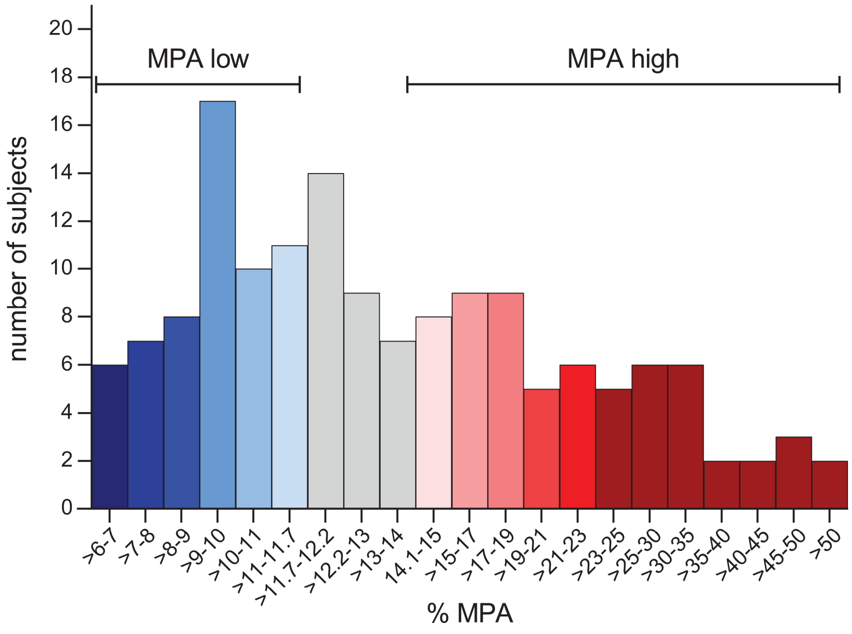

Supplemental Figure 1. MPA levels at baseline. Percentiles of monocyte platelet aggregate (MPA) levels were calculated based on MPA values of *n*=149 participants at two unique timepoints (TP). **(A)** Low MPA levels were defined as ≤40th percentile (11.7%) and high MPA levels at ≥60th percentile (14.1%).

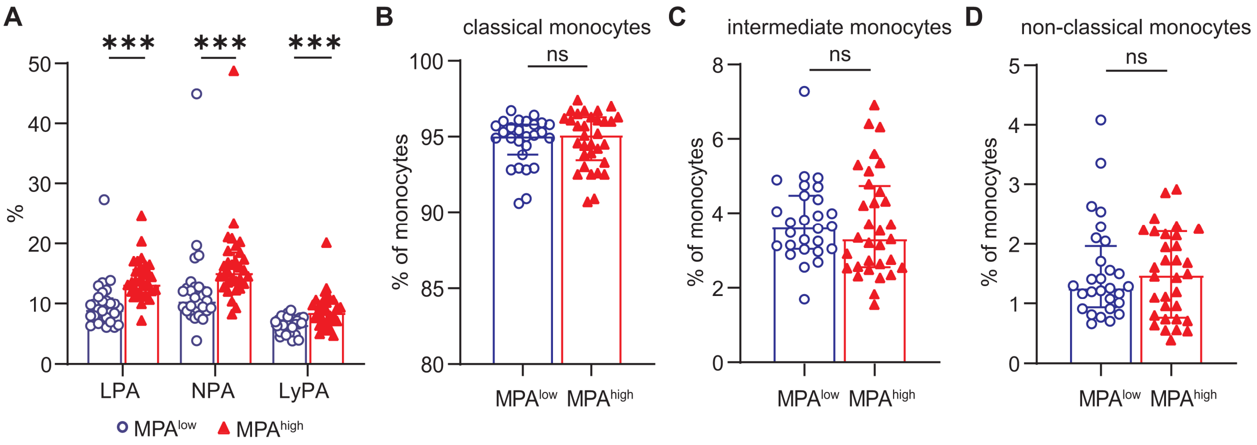

Supplemental Figure 2. Leukocyte platelet aggregates and monocyte subsets in MPA high and low groups. Whole blood was fixed with 1% formalin, stained with antibodies against CD45, C61, CD14 and CD16 and analyzed by flow cytometric analysis. **(A)** Leucocyte platelet aggregates (LPA), neutrophil platelet aggregates (NPA), and lymphocyte platelet aggregates (LyPA) were quantified based on forward side scatter properties and positivity for CD45 and CD61. **(B-D)** Monocyte subsets were defined as $CD14^{+}CD16^{-}$ classical monocytes, $CD14^{+}CD16^{+}$ intermediate monocytes and $CD14^{(+)}CD16^{+}$ non-classical monocytes. Results were compared between MPA^{high} (n=32) versus MPA^{low} (n=28) individuals. Graphs show median ± interquartile range; Man-Whitney-U test was applied, *** P<0.001.

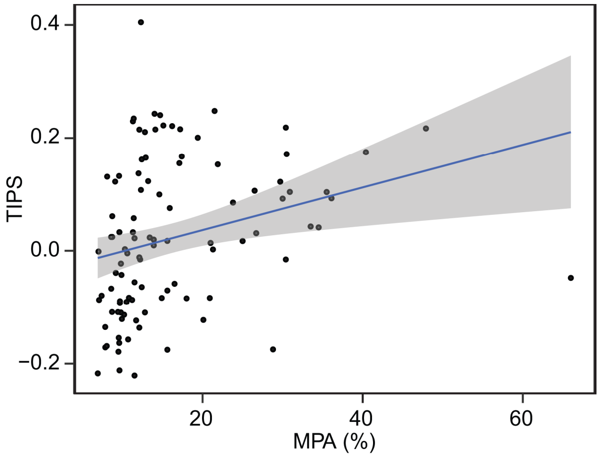

Supplemental Figure 3. Correlation of TIPS and MPA levels. Spearman's correlation of TIPS and monocyte platelet aggregate (MPA) levels in all CHORD study participants with platelet RNA sequencing and MPA levels available at time point 1 (*n*=100) (r=0.44, *P*=0.03).

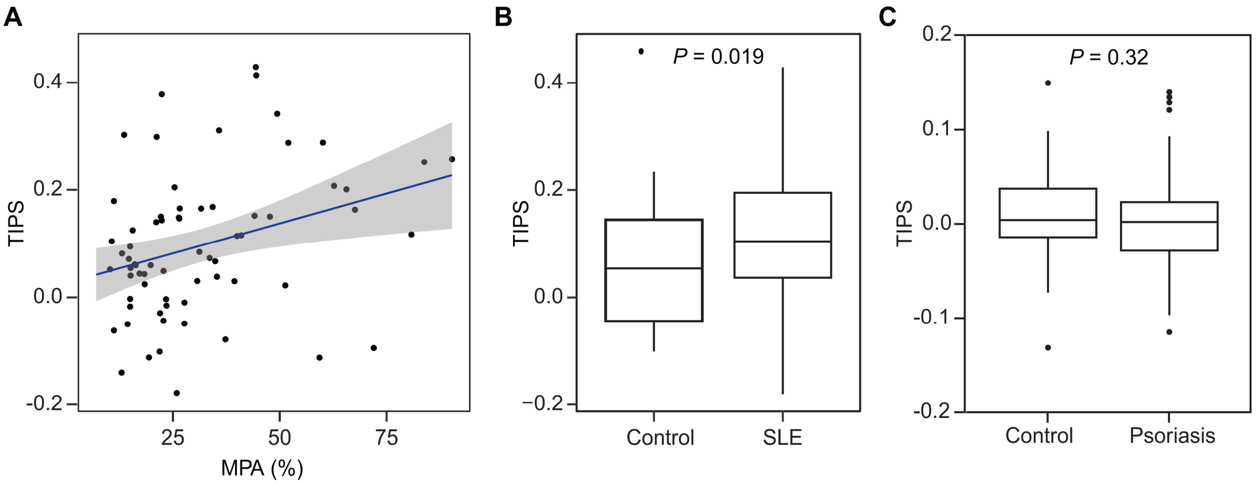

Supplemental Figure 4. TIPS in autoimmune disease cohorts. TIPS was calculated in a cohort of women with systemic lupus erythematosus (SLE) (*n*=121) and healthy controls (*n*=36) with MPA levels available for *n*=57 patients with SLE and n=12 healthy controls, and in a cohort of patients with psoriasis (*n*=52) and healthy controls (*n*=40). (**A**) Spearman's correlation of TIPS and MPA in the SLE cohort (r=0.33, *P*=0.006). (**B**) TIPS was compared between healthy controls and SLE patients and (**C**) between healthy controls and patients with psoriasis. Boxes show the interquartile range (IQR) with the median line; whiskers extend to 1.5 × IQR, points beyond that range are shown as outliers. Mann-Whitney U test was used.

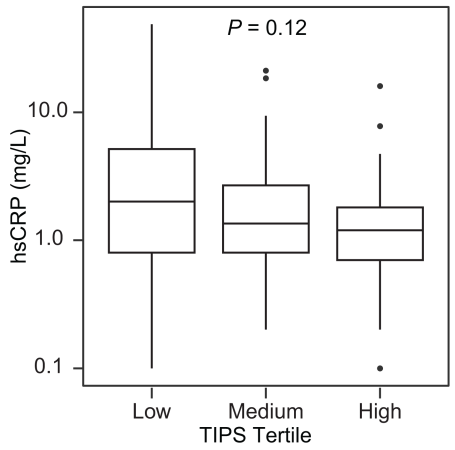

Supplemental Figure 5. Association of TIPS and high sensitive C-reactive protein. Platelet RNA sequencing for TIPS calculation and high sensitive C-reactive protein (hsCRP) were available for *n*=102 participants at timepoint 1. HsCRP was compared in participants stratified by TIPS tertile. Boxes show the interquartile range (IQR) with the median line; whiskers extend to 1.5 × IQR, points beyond that range are shown as outliers. Kruskal-Wallis test was applied.

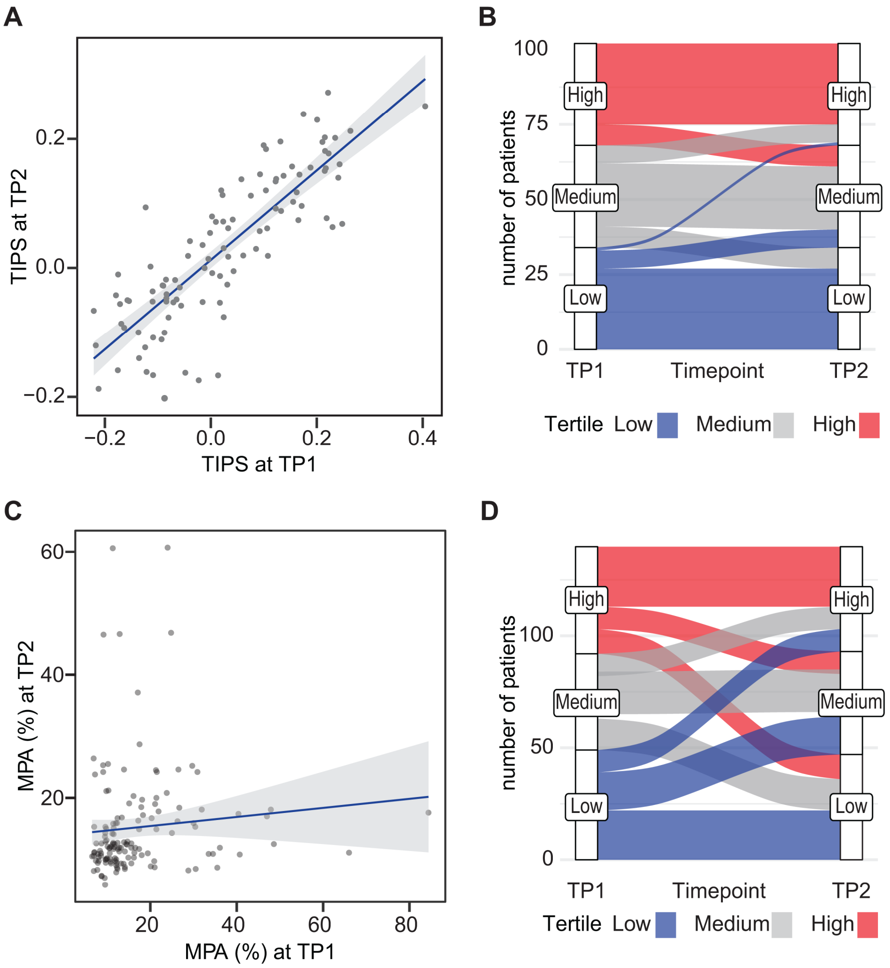

Supplemental Figure 6. Reproducibility of TIPS and circulating MPA. Platelet RNA sequencing for TIPS calculation (*n*=102) and MPA levels measured by flow cytometry (*n*=140) were available at two time points (TP) with a four-week interval. (A) Spearman's correlation of TIPS at TP1 and TP2 (r=0.84, *P*<0.001). (B) Change in TIPS tertile at TP1 and TP2. (C) Spearman correlation of MPA levels at TP1 and TP2 (r=0.25, *P*=0.003). (D) Change in MPA tertiles at TP1 and TP2.

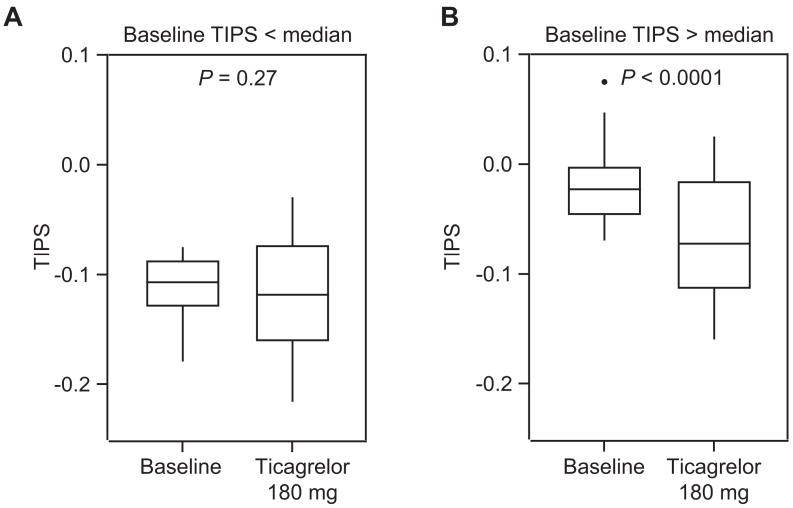

Supplemental Figure 7. Effect of ticagrelor on TIPS. TIPS was calculated in a cohort of healthy subjects that had blood collected for platelet RNA isolation at baseline (>4 weeks without antiplatelet medication) and after four weeks of treatment with 180 mg ticagrelor (*n*=50). The effects of ticagrelor on TIPS are shown for study participants with (**A**) baseline TIPS below the median and (**B**) baseline TIPS above the median. Paired t-test was applied.
